# Photocatalytic and adsorption properties of TiO_2_-pillared montmorillonite obtained by hydrothermally activated intercalation of titanium polyhydroxo complexes

**DOI:** 10.3762/bjnano.9.36

**Published:** 2018-01-31

**Authors:** Mikhail F Butman, Nikolay L Ovchinnikov, Nikita S Karasev, Nataliya E Kochkina, Alexander V Agafonov, Alexandr V Vinogradov

**Affiliations:** 1 Ivanovo State University of Chemistry and Technology, Sheremetevsky Av. 7, Ivanovo 153000, Russian Federation; 2 G.A. Krestov Institute of Solution Chemistry, Russian Academy of Sciences, Akademicheskaya St. 1, Ivanovo 153045, Russian Federation; 3 ITMO University, Lomonosova St. 9, St. Petersburg 197101, Russian Federation

**Keywords:** adsorption, intercalation, mesoporosity, photocatalytic activity, pillared montmorillonite, titanium oxide

## Abstract

We report on a new approach for the synthesis of TiO_2_-pillared montmorillonite, where the pillars exhibit a high degree of crystallinity (nanocrystals) representing a mixture of anatase and rutile phases. The structures exhibit improved adsorption and photocatalytic activity as a result of hydrothermally activated intercalation of titanium polyhydroxo complexes (i.e., TiCl_4_ hydrolysis products) in a solution with a concentration close to the sol formation limit. The materials, produced at various annealing temperatures from the intercalated samples, were characterized by infrared spectroscopy, differential scanning calorimetry (DSC)/thermogravimetric analysis (TGA), X-ray diffraction, dynamic light scattering (DLS) measurements, and liquefied nitrogen adsorption/desorption. The photocatalytic activity of the TiO_2_-pillared materials was studied using the degradation of anionic (methyl orange, MO) and cationic (rhodamine B, RhB) dyes in water under UV irradiation. The combined effect of adsorption and photocatalysis resulted in removal of 100% MO and 97.5% RhB (with an initial concentration of 40 mg/L and a photocatalyst-sorbent concentration of 1 g/L) in about 100 minutes. The produced TiO_2_-pillared montmorillonite showed increased photocatalytic activity as compared to the commercially available photocatalyst Degussa P25.

## Introduction

Titanium dioxide in its nanometer-sized form is one of the most promising modern photocatalysts [1]. However, the use of pure TiO_2_ nanoparticles is hindered by some limitations such as low adsorption capacity and possibility of particle agglomeration, which reduce its photocatalytic efficiency for the processes of purifying sewage and natural water bodies from pollutants of organic origin. To overcome these limitations, titanium dioxide nanoparticles are distributed on mineral carriers such as natural clay minerals [2], particularly montmorillonite (MM) [3]. The structure of MM is characterized by a three-layer package (2:1): two layers of silicon–oxygen tetrahedron (T) turned towards each other by their vertices, covering a layer of aluminum hydroxyl octahedra (O) on both sides. Due to isomorphous substitutions (e.g., Al^3+^ for Si^4+^ in a T layer and/or Al^3+^ for Mg^2+^ in an O layer) aluminosilicate layers are negatively charged, so positive charge compensating ions (counter ions) may move within the 2D interlayer space [4]. The weak bond between the layers results in a significant cation exchange capacity. This makes it possible to efficiently intercalate various inorganic cations into the interlayer space of MM, in particular, those possessing photocatalytic activity.

One of promising methods for modifying MM with titanium dioxide, called pillarization, consists in carrying out a reaction of exchanging the interlayer cations of MM for positively charged titanium polyhydroxo complexes or TiO_2_ sol particles and subsequent thermal treatment to yield metal oxide nanocrystals (pillars) in the interlayer space of MM [3,5]. Pillarization of MM results in an increase in the distance between aluminosilicate layers in the structure of the clay mineral and the emergence of additional micro- and mesopores. The TiO_2_-pillared MM possesses a highly developed specific surface area and improved sorptive capacity, and exhibits high activity in various photocatalytic processes, including photolysis of organic dyes [6].

The main challenge in obtaining TiO_2_-pillared MM is the synthesis of intercalants, i.e., large-size multiple charged titanium hydroxo complexes. The structure and formula for polynuclear titanium complexes is still not established exactly. For example, Einaga [7] believes that their main form is the cation (TiO)_8_(OH)_12_^4+^. However, based on an analysis of textural properties for TiO_2_-pillared MM, Bahranowski et al. [8] call this view into question by favoring polycations with higher amounts of titanium ions.

Two different methods are suggested in the literature for the synthesis of titanium polycations. The first approach is based on adding TiCl_4_ to HCl followed by dilution with distilled water [9–10]; the second is related to hydrolyzing titanium salts or alkoxides in a solution of HCl or CH_3_COOH to give a titania sol. For example, Zhang et al. [11] describes a low-temperature method for pillaring MM using a TiO_2_ sol produced by hydrolyzing TiCl_4_ in strongly acidic medium and subsequent drying at 30–80 °C. Chen et al. [12] shows the results of pillaring MM using titanium tetra-*N*-butoxide hydrolyzed in HCl solution and subsequent annealing at a temperature of 500–900 °C. The improved photocatalytic activity of the obtained TiO_2_-pillared MM in comparison with the commercial photocatalyst Degussa P 25 is revealed.

A significant advantage of using a solution prior to sol stems from the possibility of obtaining titanium hydroxo complexes with sizes as small as 1–2 nm, which favors producing pillars with adjustable size [7,12], whereas sol peptization yields particles of about 11–50 nm [13–14], i.e., considerably larger than, for example, (TiO)_8_(OH)_12_^4+^. Using a sol as a precursor often leads to the formation of a partially disordered structure in the pillar samples [15] due to (1) the nonuniform size distribution of the sol particles and (2) the formation of a partially delaminated structure [16], whereas the use of titanium hydroxo complexes ensures the formation of well-ordered structures. The use of a transparent intercalating solution makes it possible to effectively control the formation of titanium polycations by the dynamic scattering of a laser beam. Furthermore, applying titanium polycations for the MM intercalation process promotes a more uniform distribution of the precursor on the inner surface of a layered matrix. Recently, we have successfully used this advantage in the preparation of TiO_2_ particles by the biotemplate technique while impregnating cellulose with a concentrated solution of titanium polyhydroxo complexes [17].

In order to improve the textural and photocatalytic properties of pillared MM, researchers have used various intensifications at the intercalation stage, for example, performing the process in supercritical CO_2_ [18] or under the influence of microwave treatment [19–21]. As a result, it is possible to achieve increased values of specific surface area for the pillared samples and high degree of dispersion and crystallinity for TiO_2_ in the MM structure, thus enhancing photocatalytic activity. Ooka et al. [16] report some data demonstrating high efficiency of hydrothermal effects when using a TiO_2_ sol as an intercalating agent. They note that the advantage of the hydrothermal treatment stems from imparting higher acid resistance to the pillared MM, which is important when it is used for photocatalysis in highly acidic media. In particular, hydrothermal treatment accelerates crystallization and formation of clusters which grow and produce amorphous TiO_2_ nanoparticles in the interlayer space of montmorillonite prior the stage of heat treatment, and significantly improves the transformation of these particles into well-crystallized TiO_2_ pillars after further annealing. To the best of our knowledge, the literature data contain no information on hydrothermal synthesis of the pillared MM using a solution of titanium polyhydroxo complexes.

In this paper we propose and successfully apply a method for producing TiO_2_-pillared MM with a high degree of crystallinity for the TiO_2_ pillars and improved adsorption and photocatalytic activities by hydrothermally activated intercalation of titanium polyhydroxo complexes, i.e., products of a controlled TiCl_4_ hydrolysis. A solution of titanium hydroxo complexes representing the borderline between a conventional solution of a titanium salt and a TiO_2_ sol was obtained by hydrolyzing TiCl_4_ in a strongly acidic medium [9]. The effect of hydrothermal treatment and temperature on structural, textural, and adsorption properties and UV photocatalytic activity of the produced TiO_2_-pillared MM samples is assessed.

## Results and Discussion

### Structure and texture of TiO_2_-pillared materials

**Figure 1:**
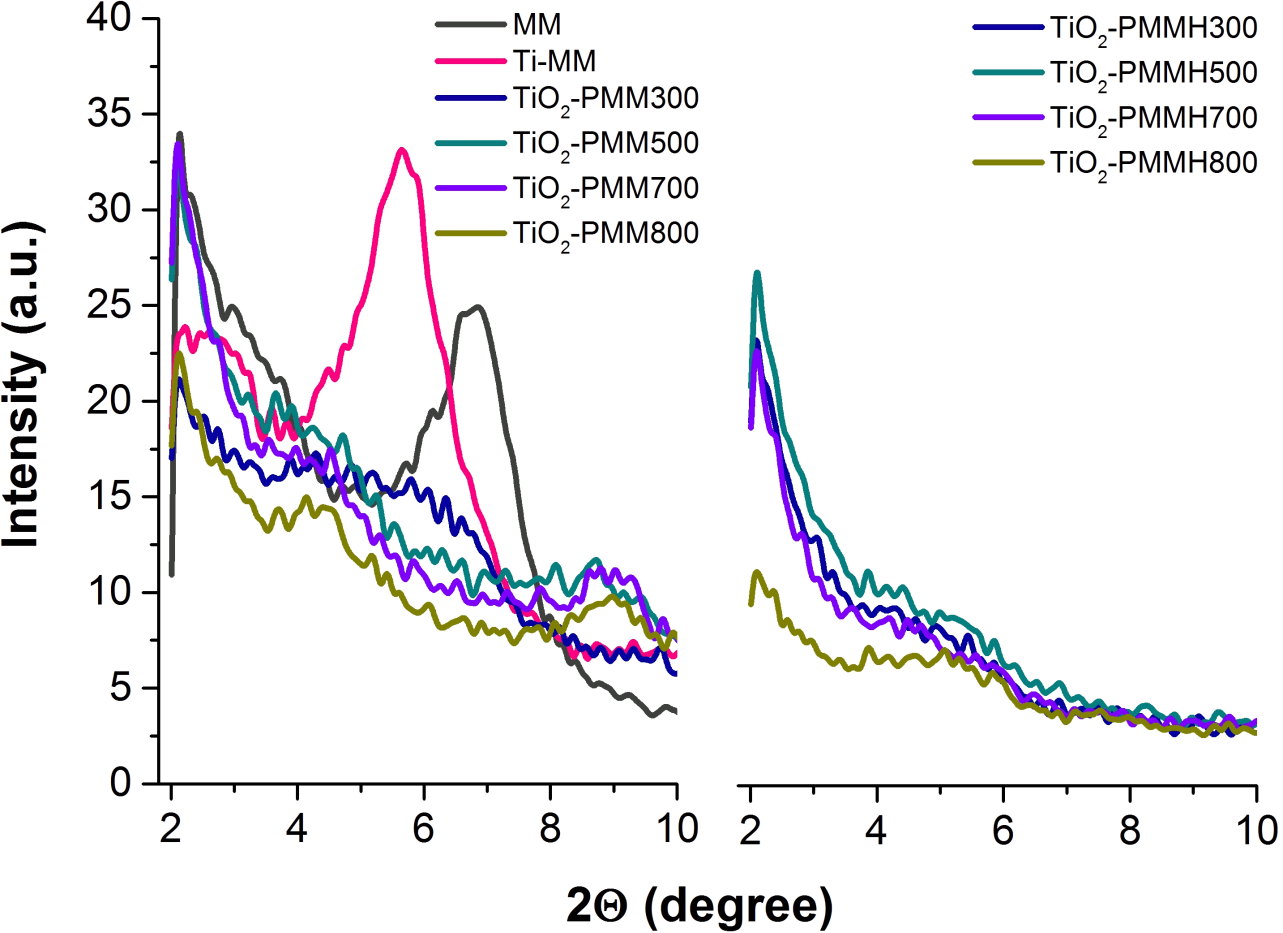
Small-angle XRD patterns of the raw MM, Ti-MM, TiO_2_-PMM*х*, and TiO_2_-PMMH*х* (see Experimental section for abbreviations).

Figure 1 shows small-angle diffractograms for the initial MM sample and that intercalated with titanium polycations, as well as for the TiO_2_-pillared materials without treatment and subjected to hydrothermal treatment (see Experimental section), calcined at different temperatures, whereas Table 1 contains their basal distances *d*_001_.

**Table 1:** Basal distance *d*_001_ of the MM, Ti-MM, TiO_2_-PMM*х*, and TiO_2_-PMMH*х* samples (see Experimental section for abbreviations).

*d*_001_, nm					

MM	Ti-MM	TiO_2_-PMM300	TiO_2_-PMM500	TiO_2_-PMM700	TiO_2_-PMM800
1.3 ± 0.01	3.8 ± 0.1	4.0 ± 0.1			
–	–	TiO_2_-PMMH300	TiO_2_-PMMH500	TiO_2_-PMMH700	TiO_2_-PMMH800
–	–	4.2 ± 0.1			

Based on an analysis of the diffraction patterns, all the TiO_2_-pillared MM samples are observed to possess well-ordered structures. As seen in Figure 1, in the case of intercalation with titanium polycations there is a shift of the characteristic peak of the original MM (2θ = 7.1°) in the direction of small angles. Upon intercalation, the basal distance *d*_001_ is increased from 1.3 nm to 3.8 nm, which indicates the penetration of polycations into the interlayer space of MM. After calcination, the *d*_001_ value (Table 1) varies from 4.0 nm for TiO_2_-PMM to 4.2 nm for TiO_2_-PMMH*x* (for abbreviations see Experimental section). High values of *d*_001_, as also observed previously in [22–23], are attributed by Sahel et al. [24] to not just simple intercalation of titanium hydroxo complexes, but also to the existence of a certain porous structure associated with the aggregation of pillars in the interlayer space of MM. Some increase in the basal distance for the hydrothermally treated samples is probably due to the processes of polymerization of the hydroxo complexes mentioned in [25–26] in the interlayer space of MM already at the stage of intercalation. It is interesting to note that the basal distance retains its value both in samples subjected to hydrothermal treatment and those without it even after annealing at 700–800 °C, indicating thermal stability of the structure of the obtained pillared materials.

**Figure 2:**
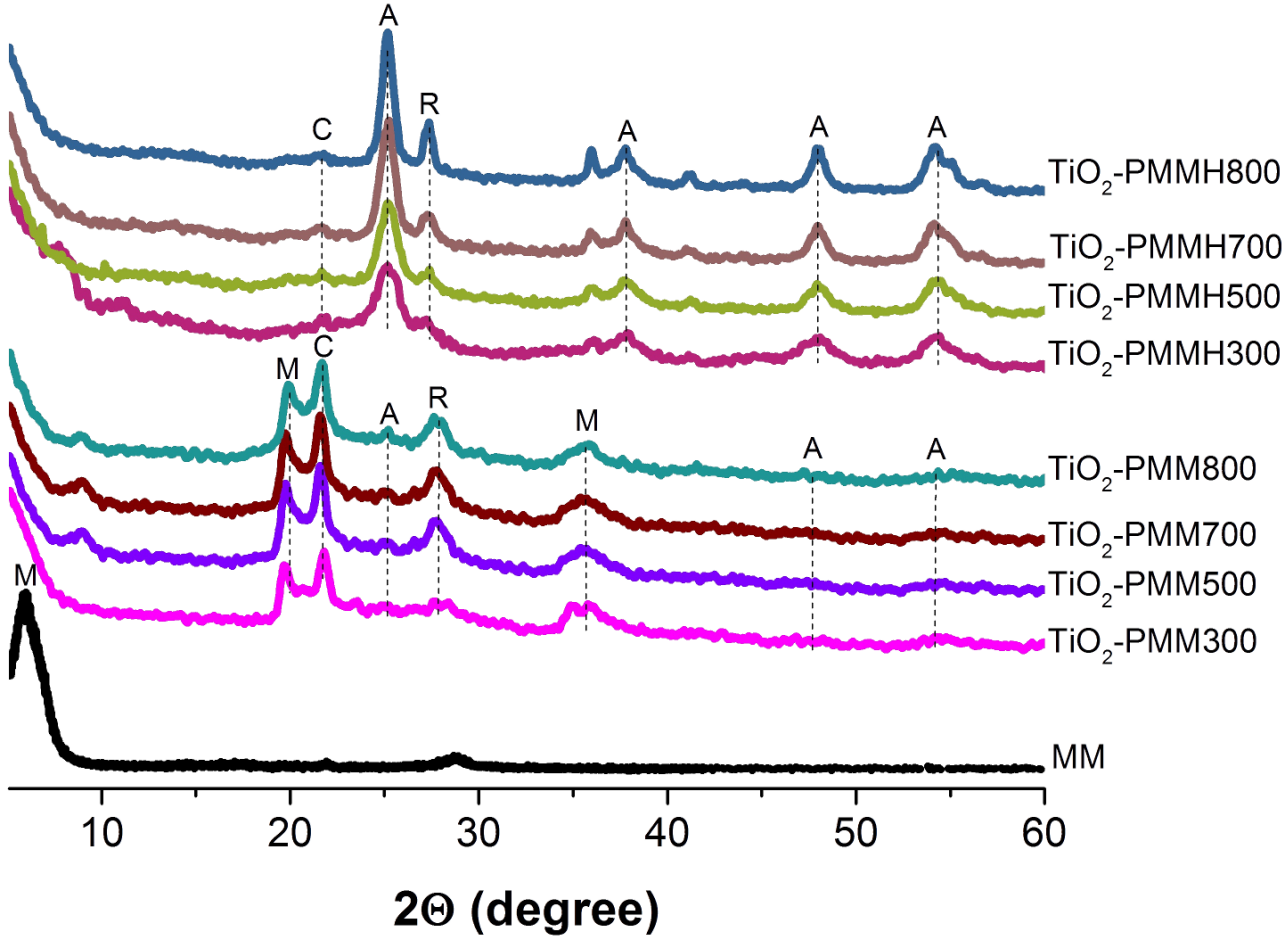
XRD patterns of the raw MM and TiO_2_-PMM*x*, and TiO_2_-PMMH*x*, where М, C, А, and R represent montmorillonite, cristobalite, anatase, and rutile phases, respectively.

Figure 2 shows the results of an X-ray analysis. One observes a single montmorillonite phase for the initial MM sample (100% intensity reflex with a basal spacing *d*_001_ of 1.3 nm). After calcination, the pillared material samples are characterized with peaks corresponding to the montmorillonite (2θ = 19.8°, 35.6°) and cristobalite (2θ = 21.7°) phases. The cristobalite phase which has also been observed in [27], is a secondary product probably produced under the synthesis conditions with a low pH value of the medium. The diffractograms of the TiO_2_-pillared samples calcined at 300 °C have shown the absence of the anatase phase, and from 500 °C broad and weak peaks of both anatase (at 2θ = 25.3°, 47.8°, 54.4°) and rutile (at 2θ = 27.7°) could be identified, which is in agreement with the data of [28] where the co-presence of both anatase and rutile phases at calcination temperatures of 450–500 °C has been established.

The TiO_2_-PMMH*x* samples (Figure 2) showed significantly more sharp and intense peaks of the anatase and rutile phases at the above mentioned angles. In addition, the anatase peak becomes visible at 2θ = 37.7°. These results demonstrate that in the preparation of the pillared materials employing hydrothermal treatment, the TiO_2_ pillars crystallize in the anatase and rutile phases much more readily than in the absence of that treatment.

The mass fraction of rutile crystallites (*X*_R_) in the samples of TiO_2_-pillared materials was determined from the radiographs by the ratio of heights of peaks (101) of anatase *I*_A_ and (110) of rutile *I*_R_ according to the equation [29]:

[1]
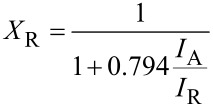


The hydrothermally intercalated samples annealed at 300–800 °C are a mixture of the anatase and rutile phases in a ratio that ranges from 86:14 to 72:28 with increasing calcination temperature (Table 2).

**Table 2:** Average crystallite size and phase composition of the TiO_2_-PMMН*x* samples (see Experimental sections for a description of the sample names).

Sample	Average crystallite size, *L* (nm)	Phase composition (%)	
		Anatase	Rutile

TiO_2_-PMMН300	5.6	86	14
TiO_2_-PMMН500	7.3	80	20
TiO_2_-PMMН700	9.1	77	23
TiO_2_-PMMН800	12.7	72	28

As seen in Figure 2, the intensity of peaks for anatase and rutile increases with increasing calcination temperature. This suggests that higher temperatures can promote the growth of crystallites. Indeed, the size of the anatase crystallites sequentially increases from 5.6 to 12.7 nm (Table 2) with increasing calcination temperature from 300 to 800 °C. According to the data of Figure 2, the phase transformation of anatase into rutile lies in the temperature range from 500 to 800 °C.

**Figure 3:**
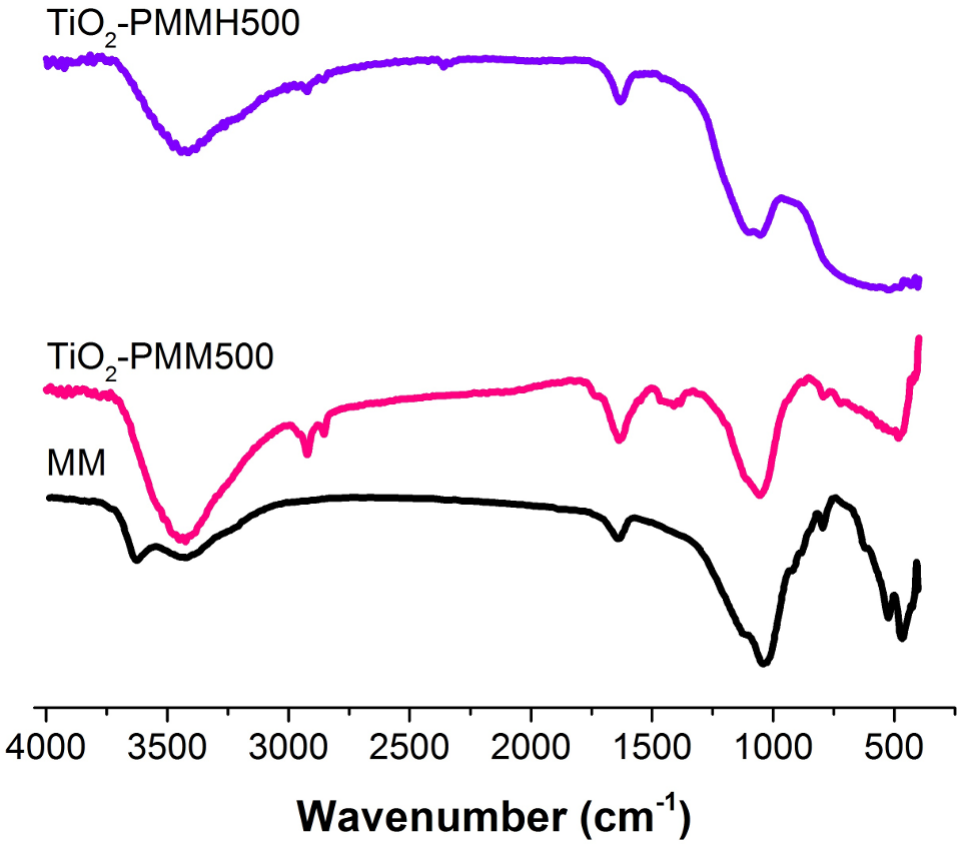
FTIR spectra of the raw MM, and TiO_2_-pillared MM.

Figure 3 shows the IR spectra of the original MM, TiO_2_-PMM500 and TiO_2_-PMMH500 samples as an example (because of their identity) of a TiO_2_-pillared material. Spectra of all the samples in the region 3700 to 2800 cm^−1^ feature bands due to stretching vibrations of the OH groups. In particular, a wide band centered at about 3440 cm^−1^ is associated with the OH groups of the layered structure [30], which is even further broadened in the spectra of pillared samples. Low-intensity absorption bands at 2920 and 2850 cm^−1^ observed in the spectra of samples TiO_2_-PMM500 and TiO_2_-PMMH500 is attributed, according to [31], to the isolated OH groups on the surface of TiO_2_.The absorption band at 1636 cm^−1^ in the region 1700 to 1500 cm^−1^ results from bending vibrations of the OH groups of adsorbed water molecules. Note that for all the samples of pillared materials the intensity of this band compared to the original MM increases due to the conservation of a larger amount of water molecules during the formation of TiO_2_, as noted previously in [32]. The intense band between 800 and 1200 cm^−1^ centered at about 1040 cm^−1^ corresponds to the Si–O stretching vibrations [30,33]. A change in its intensity indicates the formation of cross-links of pillars with silicate layers. Changes in the spectra of the pillared samples between 930 and 400 cm^−1^ may be caused by the formation of both the Si–O–Ti bonds, yielding absorption bands at 920, 780, and 725 cm^−1^ [34], and the Ti–O and Ti–O–Ti bonds, absorption bands of which are located in the region 800 to 400 cm^−1^ [35–36]. Based on an analysis of the IR spectra one should expect that the increased hydroxylation of the surface of pillared MM contributes to increasing adsorption of the reactant and its delivery to the active photocatalytic centers of titanium dioxide.

**Figure 4:**
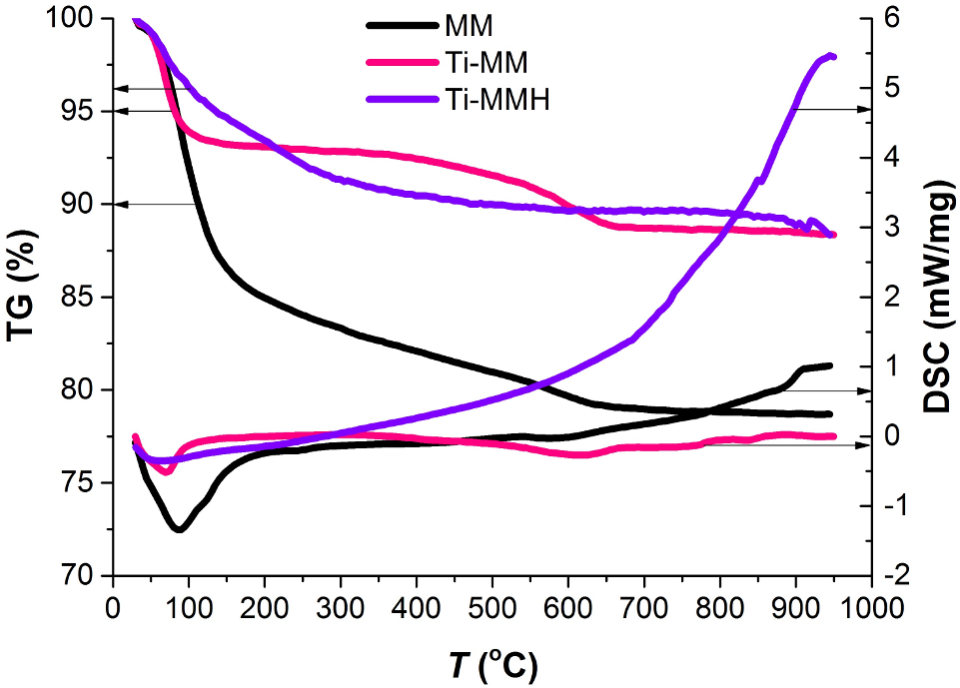
Thermogravimetric analysis (TGA)/differential scanning calorimetry (DSC) curves of the raw MM, Ti-MM, and Ti-MMH.

The results of a synchronous (thermogravimetric analysis (TGA) and differential scanning calorimetry (DSC)) thermal analysis of the samples are shown in Figure 4.

As seen in Figure 4, the MM, Ti-MM and Ti-MMH samples yield similar thermogravimetric curves, in which the loss of mass for all the samples occurs in two stages [37]. The first stage is observed in the temperature range 70–150 °C (first endothermic effect) and accompanied with 6.7, 17.1, and 6.6% weight losses for the samples MM, Ti-MM, and Ti-MMH, respectively. This weight loss is associated with the release of physically adsorbed water from the interlayer space. Further reduction in mass (4.0, 6.7, and 3.7% for the MM, Ti-MM, and Ti-MMH, respectively) in the range from 260 to 580 °C corresponds to the dehydroxylation of titanium polycations located in the interlayer space of MM, as well as aluminosilicate layers (second endothermic effect). The total weight loss in air-dry conditions for Ti-MM (23.8%) increased compared to the original MM (10.7%) due to the dehydration of intercalated polyhydroxo complexes. However, in the case of Ti-MMH there is a relatively low total weight loss (10.3%) compared to Ti-MM, which is probably a result of the aggregation of hydroxo complexes under hydrothermal conditions.

Data on the zeta potentials for the particles of all samples dispersed in double distilled water are shown in Table 3.

**Table 3:** Average values of the zeta potential for the samples tested in this work.

Sample	Zeta potential, mV

ММ	−29.1
Ti-MM	−24.2
TiO_2_-PMM300	−26.4
TiO_2_-PMM500	−35.7
TiO_2_-PMM700	−33.9
TiO_2_-PMM800	−31.5
TiO_2_-PMMH300	−5.3
TiO_2_-PMMH500	−16.3
TiO_2_-PMMH700	−16.0
TiO_2_-PMMH800	−17.8

The value of this parameter is often used as a measure of attractive/repulsive electrostatic interaction forces between the particles and provides information on the charge state of the surface of particles in the suspension. It is worth noting that all the samples possess negative values of the zeta potential in water. As is evident from Table 3, the zeta potential values for TiO_2_-PMM*x* ranged from −26.4 to −31.5 mV. The TiO_2_-PMMH*x* samples show a decrease in the magnitude of the zeta potential. This result is probably related to the formation of larger particles of TiO_2_ on the external surface of MM due to flocculation at high titanium content, which leads to a reduction in their electrophoretic mobility.

**Figure 5:**
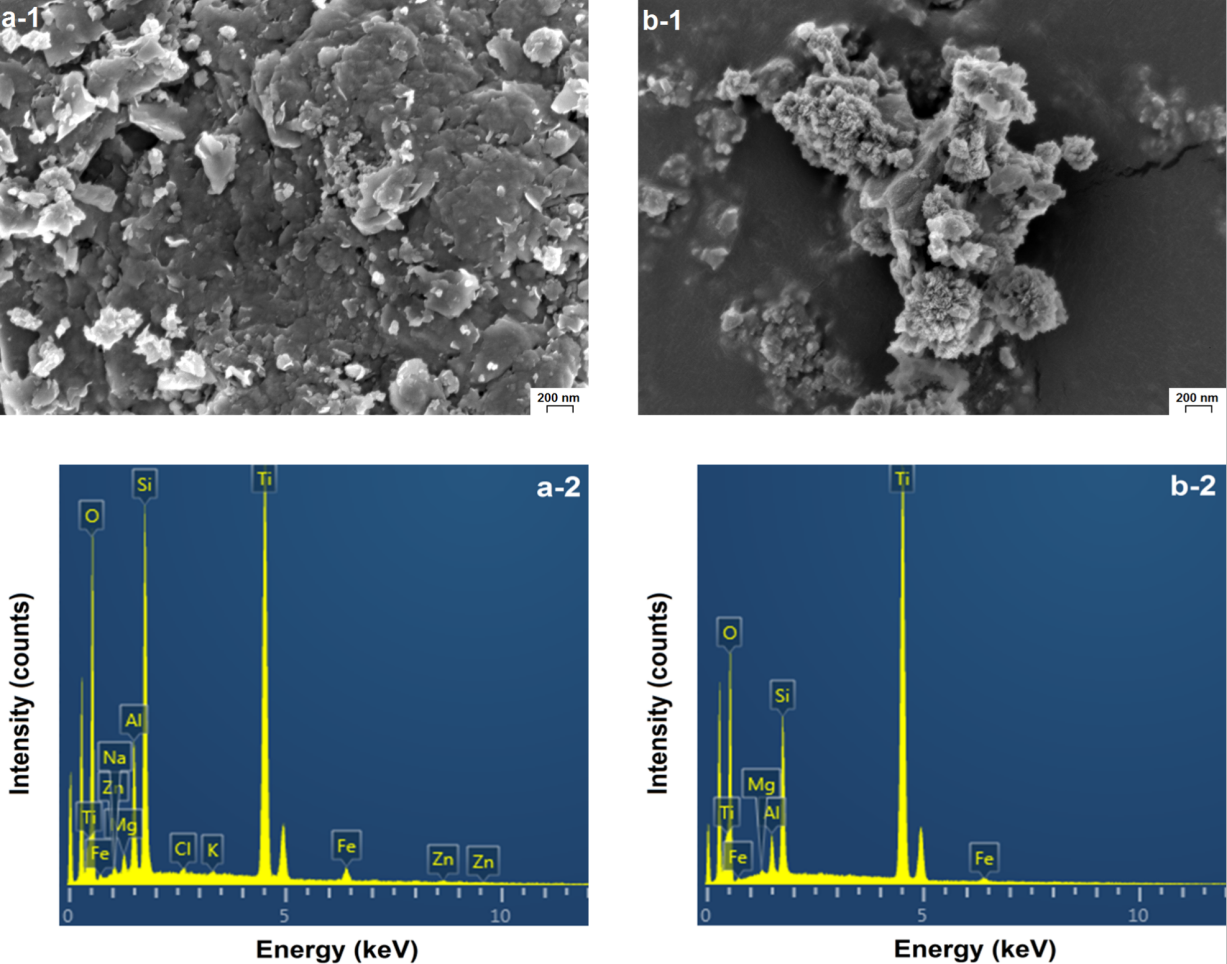
SEM images and EDS spectra of the surface of (a) TiO_2_-PMM500, and (b) TiO_2_-PMMH500.

In pillared samples, titanium oxide may probably exist both in the interlayer space and on the external surface of pillared MM. Figure 5 shows SEM images of the pillared samples TiO_2_-PMM500 and TiO_2_-PMMH500. Their morphologies have essential differences. In the case of TiO_2_-PMM500 (Figure 5a-1), agglomerates of TiO_2_ crystallites with different shapes and sizes dispersed on the surface of MM are clearly seen. On the surface of TiO_2_-PMMH500 (Figure 5b-1) there are coral-like formations of titanium oxide, between which the TiO_2_ aggregates are distributed. Such a surface profile of a hydrothermally treated sample is consistent with its increased porosity (see Table 5 below). The fact of increasing the amount of titanium on the surface of hydrothermally treated pillared samples compared to the samples without treatment is confirmed by the results of an elemental analysis using EDS spectra (Figure 5a-2 and 5b-2). These results are shown in Table 4.

**Table 4:** Elemental analysis of the MM, TiO_2_-PMM500, and TiO_2_-PMMН500 samples.

Sample	Element, wt %										
	O	Na	Mg	Al	Si	Cl	K	Ca	Ti	Fe	Zn

MM	64.26	1.43	2.06	6.59	21.19	0.18	0.16	0.51	0.30	3.31	–
TiO_2_-PMM500	55.16	0.32	0.67	3.79	9.99	0.22	0.14	–	27.58	1.61	0.51
TiO_2_-PMMН500	56.47	–	0.19	1.47	5.58	–	–	–	35.75	0.54	–

It is quite difficult to obtain quantitative information on the content of TiO_2_ in the interlayer space and on the external surface of pillared MM. However, given that the specific surface area of intraporous space for pillared MM (*S*_int_ > 200 m^2^/g, the BET method) is many times larger than the area of its external surface (*S*_ext_ = 0.5 m^2^/g, the Tovarov method [38]), it is reasonable to assume that TiO_2_ will be predominantly in the form of pillars in the interlayer space.

**Figure 6:**
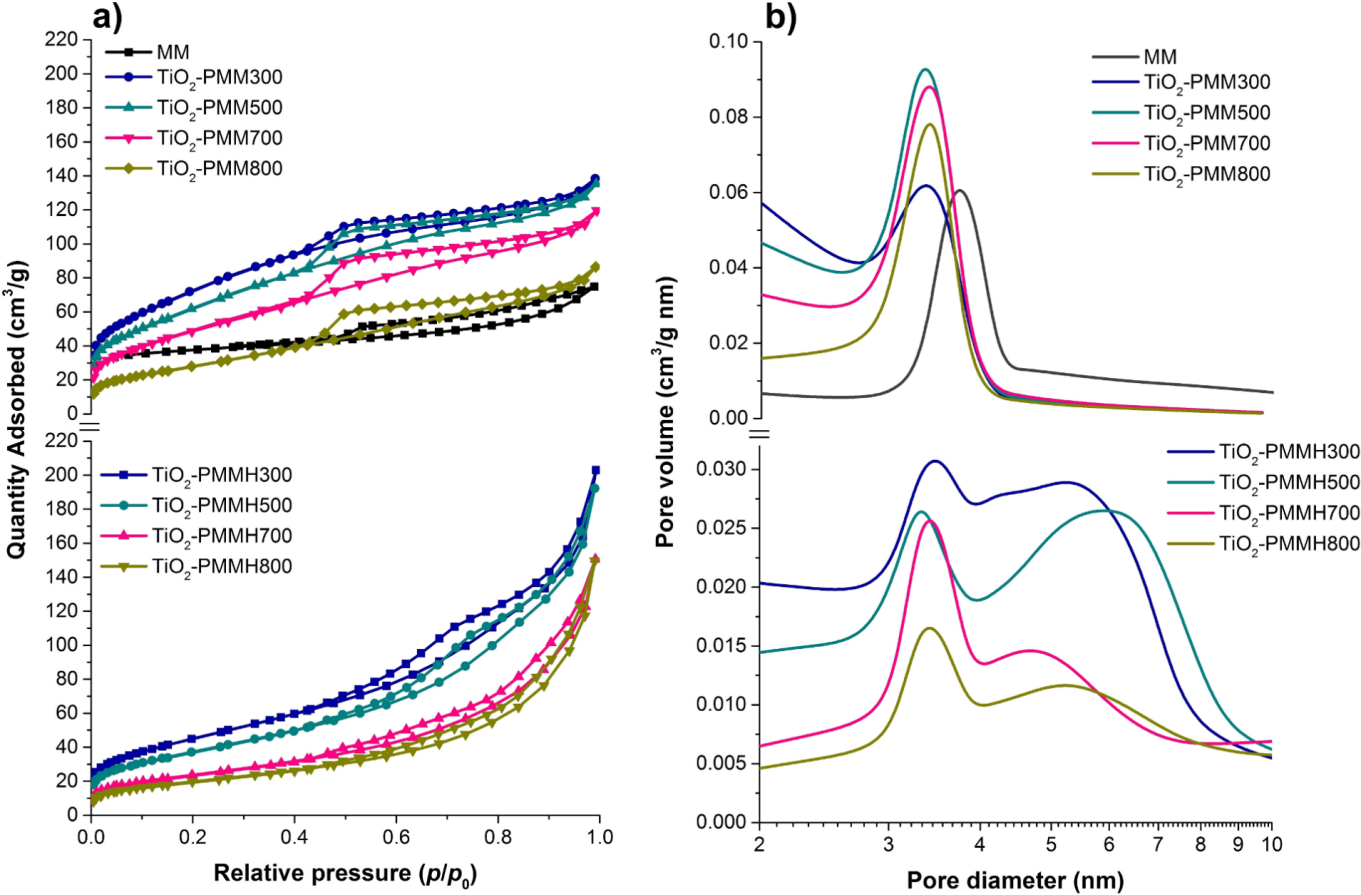
Nitrogen adsorption/desorption isotherms (а) and pore size distribution (b) of the raw MM, TiO_2_-PMM*x*, and TiO_2_-PMMH*x*.

Low-temperature nitrogen adsorption–desorption isotherms for the obtained samples are shown in Figure 6a.

All the isotherms are characterized by the presence of capillary condensation hysteresis loops and belong to type IV according to IUPAC classification [39], which is typical for materials with mesoporous structure. Furthermore, the shape of the hysteresis loop for these isotherms is of type H3 according to IUPAC [34], indicating the presence of long slit-shaped and plane-parallel pores in the obtained pillared materials. In addition, the desorption branch of the isotherm shows an inflection at about 0.45–0.5 *P*/*P*_0_, typical for many types of layered materials while using nitrogen as an adsorbent [40–41]. At *P*/*P*_0_ ratios close to 1 the isotherms of the samples TiO_2_-PMMH*x* feature a sharp rise of the sorption curve indicating the presence of large pores in the pillared samples, which is confirmed by the average pore diameter data (*D*_av_) (Table 5).

**Table 5:** Textural characteristics of TiO_2_-pillared MM samples.

Sample	*S*_BET_, m^2^/g	∑*V*_pore_, cm^3^/g	*D*_av_, nm

MM	96.0	0.170	7.00
TiO_2_-PMM300	258.2	0.214	3.79
TiO_2_-PMM500	228.5	0.209	3.98
TiO_2_-PMM700	177.3	0.185	4.41
TiO_2_-PMM800	106.1	0.134	5.02
TiO_2_-PMMН300	161.4	0.320	7.85
TiO_2_-PMMН500	135.1	0.303	8.34
TiO_2_-PMMН700	85.4	0.235	10.09
TiO_2_-PMMН800	71.5	0.234	11.72

The values of specific surface area according to Brunauer–Emmett–Teller (BET) (*S*_BET_) and total pore volume (Σ*V*_pore_) (by the Barrett–Joyner–Halenda (BJH) method) are shown in Table 5, revealing that the process of pillarization leads to significant increases in specific surface area and total pore volume compared to the original MM.

As seen from Table 5, the use of hydrothermal treatment causes a decrease in specific surface area and an increase in total pore volume in the TiO_2_-PMMH*x* samples.

Increasing calcination temperature from 300 to 800 °C leads to a decrease in specific surface area and pore volume for all pillared samples (Table 5). However, calcination at 800 °C leads to a drastic decrease in specific surface area, which apparently reflects a partial collapse of the pillared structure. Increasing the temperature from 300 to 800 °C causes a gradual increase in *D*_av_ in the TiO_2_-PMM*x* samples. A similar increase was also observed in [42] and related to the growth of TiO_2_ anatase crystals.

The pore size distribution curves are shown in Figure 6b. The distribution pattern for the TiO_2_-PMM*x* samples is narrow and unimodal. The samples prepared using hydrothermal treatment feature a shift of the distribution curve to larger pore sizes and the broadening of its shape. Furthermore, as seen in Figure 6b, the TiO_2_-PMMH*x* samples already demonstrate two peak sat 300 and 500 °C, and 3 peaks above 700 °C. This is due to the aggregation processes in the interlayer space of MM activated under hydrothermal conditions. The phenomenon of pillar enlargement can also be explained by Ostwald ripening [43]. Pillar growth occurs due to recondensation, i.e., via the process of dissolving relatively small TiO_2_ pillars in water followed by re-precipitation at high pillars.

**Figure 7:**
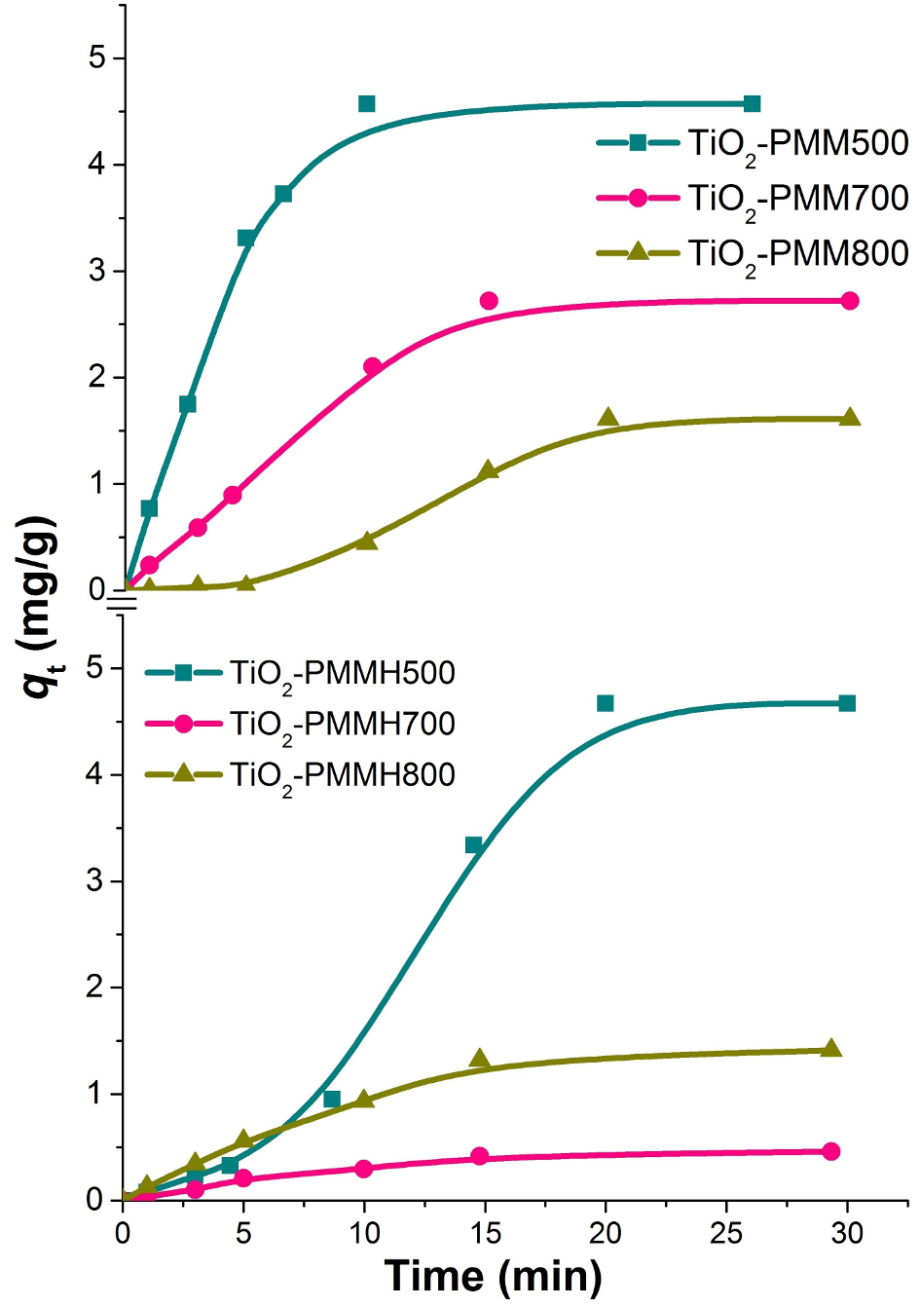
Kinetic curves of methyl orange dye adsorption at 20 °C by the TiO_2_-PMM*x*, and TiO_2_-PMMН*x* samples.

**Figure 8:**
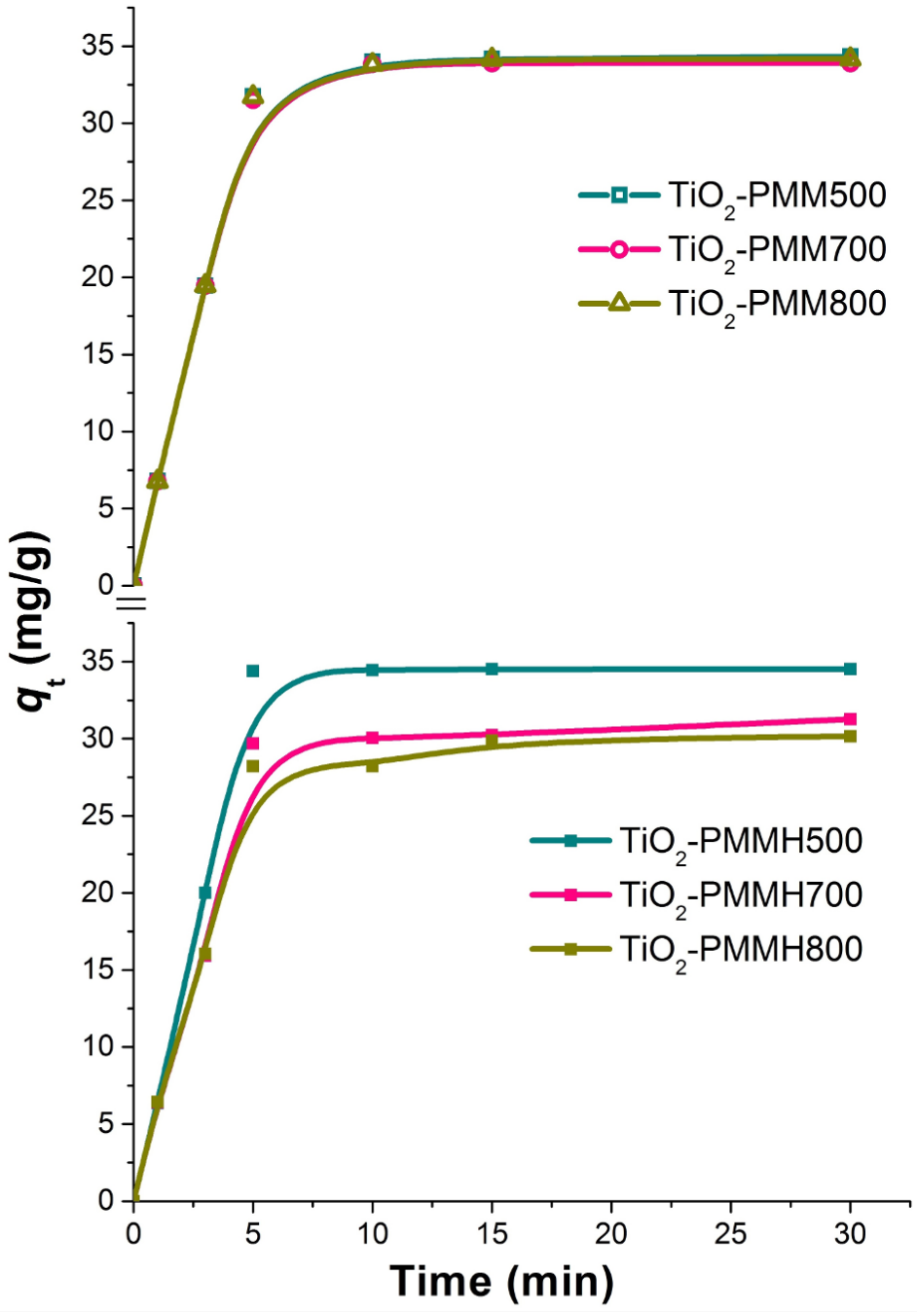
Kinetic curves of rhodamine B dye adsorption at 20 °C by the TiO_2_-PMM*x*, and TiO_2_-PMMН*x* samples.

The effectiveness of decolorization for the solutions of model dyes (anionic type MO and cationic type RhB) was assessed by measuring a decrease in concentration in the dark and under UV irradiation. It is known that the efficiency of dye removal from aqueous solutions on TiO_2_-pillared MM is determined by the additive process of photolysis, adsorption, and photocatalysis [37]. Therefore, we studied the kinetics of adsorption for the MO and RhB dyes (Figure 7, Figure 8) first.

All the samples under study reach adsorption equilibria in about 30 minutes. Adsorption rate for the cationic RhB is significantly higher than that for the anionic MO due to electrostatic interaction (see Table 3). The TiO_2_-PMM500 and TiO_2_-PMMH500 samples demonstrated maximum adsorption abilities of 4.6 mg/g and 34.5 mg/g, respectively, with respect to MO and RhB at removal efficiencies of approximately 14% and 87%. It is interesting to note that TiO_2_-PMM500 and TiO_2_-PMMH500 had almost identical adsorption efficiencies with respect to both dyes, although their textural characteristics (Table 5) are significantly different. Apparently, a decrease in specific surface area is canceled for this process by an increase in pore volume and size.

When increasing calcination temperature to 700 and 800 °C, one observes a decrease in adsorption abilities of the samples of pillared materials for both dyes, and for the samples obtained using hydrothermal treatment this decrease is faster, which correlates with the results of studying textural characteristics (Table 5).

In order to describe kinetics of the process of adsorbing model anionic and cationic dyes from aqueous solutions on TiO_2_-pillared MM, we used well-known pseudo-first order kinetic models of Lagergren [44], pseudo-second order models by Ho and McKay [45], and intraparticle diffusion models by Morris and Weber [46] and others [47], which may be respectively represented by the following equations:

[2]
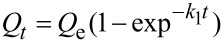


[3]
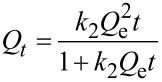


[4]
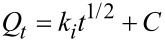


where *Q_t_* and *Q*_e_ are the amounts of adsorbed dye per unit weight of adsorbent at a given time *t* (mg/g) and at equilibrium, respectively; *k*_1_ and *k*_2_ are the adsorption rate constants of pseudo-first (min^−1^) and pseudo-second order (mg g^−1^ min^−1/2^), respectively; *k*_i_ is the constant of intraparticle diffusion rate (mg g^–1^ min^–1/2^), *t* is the phase contact time (min), *C* is an y-intercept for the *Q_t_* = *f*(*t*^1/2^) function.

The approximation and calculation of kinetic model parameters were performed using the Origin 7.5 software package (OriginLab, USA) with a nonlinear least-squares method. The determination coefficient *R*^2^ served as a criterion for the adequacy of kinetic models. The results of the nonlinear approximation of the experimental data are shown in Figure 7 and Figure 8; the model parameters are given in Table 6.

**Table 6:** Parameters of methyl orange (MO) and rhodamine B (RhB) dye adsorption kinetics for the obtained TiO_2_-pillared MM samples.

Kinetic model	Adsorbents			
	TiO_2_-PMM500		TiO_2_-PMMН500	

Pseudo-first order	MO	RhB	MO	RhB
*Q*_e_ (mg g^−1^)	4.81	35.20	7.61	35.68
*k*_1_ (min^−1^)	0.218	0.312	0.034	0.329
*R*^2^	0.982	0.973	0.891	0.953
Pseudo-second order				
*Q*_e_ (mg g^−1^)	5.81	40.63	24.75	41.05
*k*_2_ (g mg^−1^ min^−1^)	0.040	0.009	0.036	0.010
*R*^2^	0.952	0.940	0.898	0.912
Intraparticle diffusion				
*k*_i,_ (mg g^−1^ min^−1/2^)	1.023	6.810	1.047	6.853
*C* (mg g^−1^)	0.381	5.914	1.037	6.345
*R*^2^	0.792	0.702	0.828	0.656

The obtained *R*^2^ values indicate the applicability of kinetic models of pseudo-first and pseudo-second order to describe the adsorption kinetics for MO and RhB on TiO_2_-pillaed MM. Preference should be given to the pseudo-first order kinetic model, which indicates that adsorption is preceded by diffusion. The process of adsorption for both dyes on TiO_2_-pillared samples is likely to include both physical and chemical sorptions [48]. Given a high degree of adequacy of the pseudo-first order model, one can conclude that the process is significantly affected by film diffusion, and we are dealing with a high initial concentration of sorbate [49].

**Figure 9:**
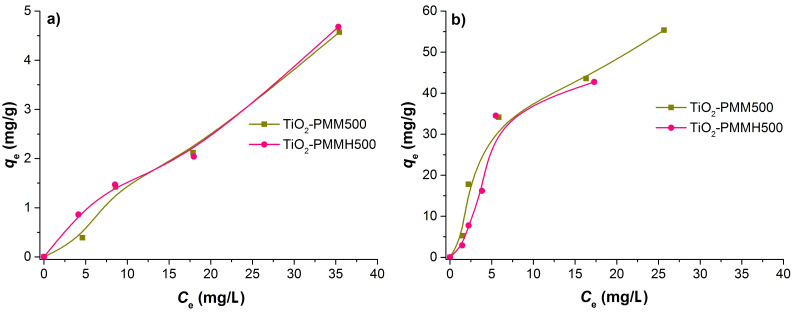
Adsorption isotherms of (a) MO and (b) RhB dyes at *T* = 20 °C.

Figure 9 represents the isotherms of MO and RhB adsorption on TiO_2_-PMM500 (Figure 9a) and TiO_2_-PMMH500 (Figure 9b), which are related an adsorption model fitting the experimental data in the most appropriate way.

Thereby, the hydrothermal treatment does not significantly influence the adsorption capacity. The curve shape in Figure 9 corresponds the L-type adsorption isotherm, according to the classification of Giles, Smith, and Huitson [50].

Besides, the adsorption isotherms were analysed by the Freundlich, Langmuir, and Sips models [51–52], represented mathematically as follows:

[5]
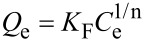


[6]
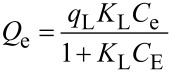


[7]
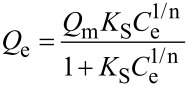


where *C*_e_ (mg/L) is the equilibrium liquid phase concentration of the adsorbate; *Q*_e_ (mg/g) the equilibrium solid phase concentration of adsorbate; *q*_L_ (mg/g) is the maximum sorption capacity; *K*_L_ (L/mg) and *K*_F_ (mg/g) are the Langmuir and Freundlich constants, respectively; *Q*_m_ (mg/g) and *K*_S_ (L/mg) are the Sips constants; 1/n is the empirical parameter. The adsorption parameters and correlation coefficients *R*^2^ are represented in Table 7.

**Table 7:** Freundlich, Langmuir and Sips constants for MO and RhB adsorption.

	Adsorbents			
	TiO_2_-PMM500		TiO_2_-PMMН500	

Freundlich	MO	RhB	MO	RhB
*K*_F_	0.13	11.08	0.18	7.51
n	1.00	0.50	0.91	0.63
*R*^2^	0.987	0.948	0.977	0.849
Langmuir				
*q*_L_ (mg g^−1^)	4.90	67.47	2.33	72.87
*K*_L_ (L mg^−1^)	0.070	0.139	0.381	0.092
*R*^2^	0.820	0.968	0.467	0.891
Sips				
*Q*_m,_ (mg g^−1^)	4.73	56.31	2.70	43.91
*K*_S_ (L mg^−1^)	0.068	0.114	0.687	0.010
n	1.01	1.42	0.88	3.27
*R*^2^	0.819	0.974	0.443	0.982

According to the *R*^2^ values, the Freundlich model describes MO adsorption more adequately, whereas in the case of RhB, the Sips model is more suitable.

**Figure 10:**
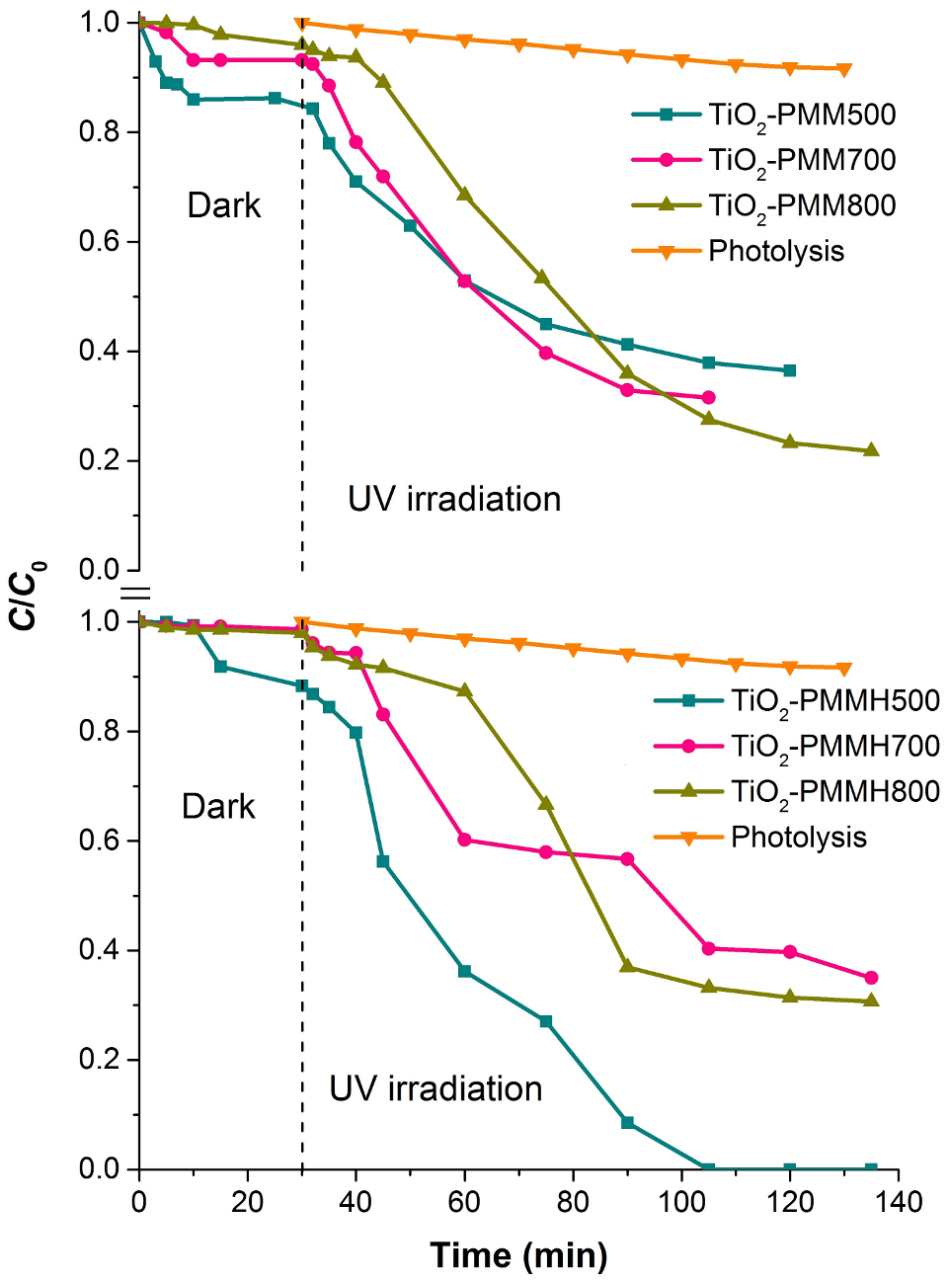
Removal of methyl orange (MO) on the TiO_2_-PMM*x*, TiO_2_-PMMН*x* samples used as photocatalysts and that without photocatalyst in the dark and under UV irradiation.

**Figure 11:**
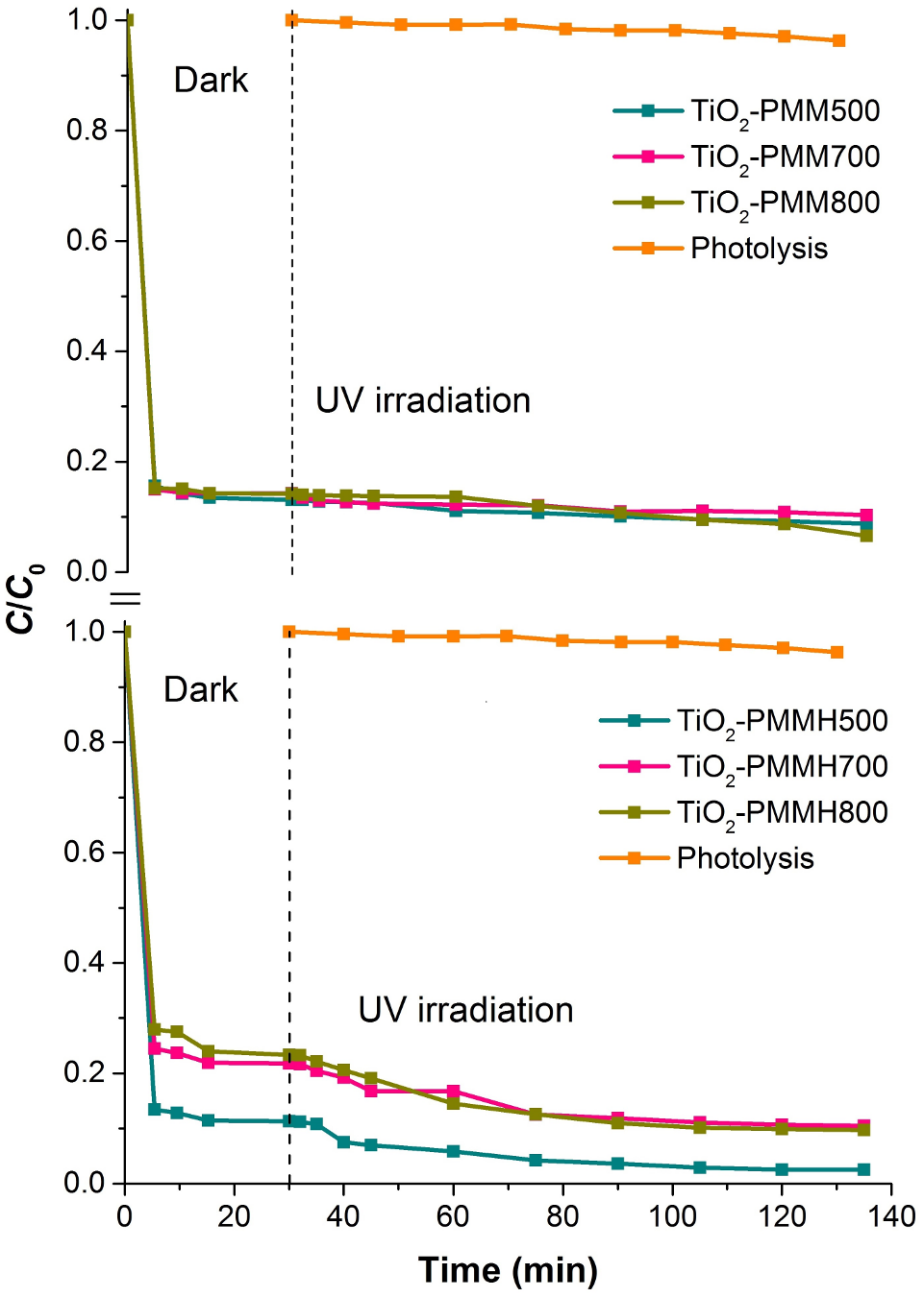
Removal of RhB on the TiO_2_-PMM*x*, TiO_2_-PMMН*x* samples used as photocatalysts and those without photocatalyst under dark and under UV irradiation conditions.

The results for the removal of the MO and RhB dyes in aqueous solution in the presence of produced photocatalysts under UV irradiation are shown in Figure 10 and Figure 11.

These results indicate that the degradation of the MO and RhB dyes upon irradiation for 1 hour in the absence of photocatalysts was only 8% and 4%, respectively, thus that process can be neglected. Prior to obtaining the kinetic curves of photodegradation for the MO and RhB solutions, the photocatalyst samples were exposed to solutions of respective dyes with constant stirring in the dark for 30 min to reach adsorption–desorption equilibrium.

The photocatalytic effect and adsorption for the two types of dyes are substantially different. For example, a total MO removal efficiency of 64% by the TiO_2_-PMM500 sample breaks down into 14% adsorption and 86% photocatalytic decomposition, whereas in the case of removing RhB a total efficiency of 91% breaks down into 87% adsorption and 13% photocatalytic decomposition.

Upon increasing calcination temperatures in the photocatalyst series TiO_2_-PMM500, TiO_2_-PMM700, TiO_2_-PMM 800 (Figure 10 and Figure 11) the rate of removing MO and RhB sequentially increases, reaching a maximum value for TiO_2_-PMM800 (79% and 94%, respectively), and the lowest efficiency among all photocatalysts under study was shown by the TiO_2_-PMM500 sample, which removed about 62% MO and 89% RhB in 120 minutes. The photocatalyst series TiO_2_-PMMH500, TiO_2_-PMMH700, TiO_2_-PMMH800 features a reverse trend – removal rate decreases for both dyes. The TiO_2_-PMMH500 sample removes virtually 100% MO and 97.5% RhB from solution in 100 minutes.

The considerably higher decolorization rate in this case can be explained by the higher degree of crystallinity for pillars and higher porosity (Table 5), which is known to greatly affect the performance of the catalyst [53]. In addition, anatase and rutile phase ratio can play an important role, which, as noted in [54], can significantly affect the rate of photocatalytic decomposition. While interpreting the photocatalytic activity of TiO_2_-pillared clays one should keep in mind the formation of cross-links between pillars and silicate layers to give the Ti–O–Si bonds, which constrain the electron–hole recombination [55]. According to this logic, the larger the crystallites of TiO_2_, the greater the number of cross-linking bonds and the higher the photocatalytic activity of pillars.

**Figure 12:**
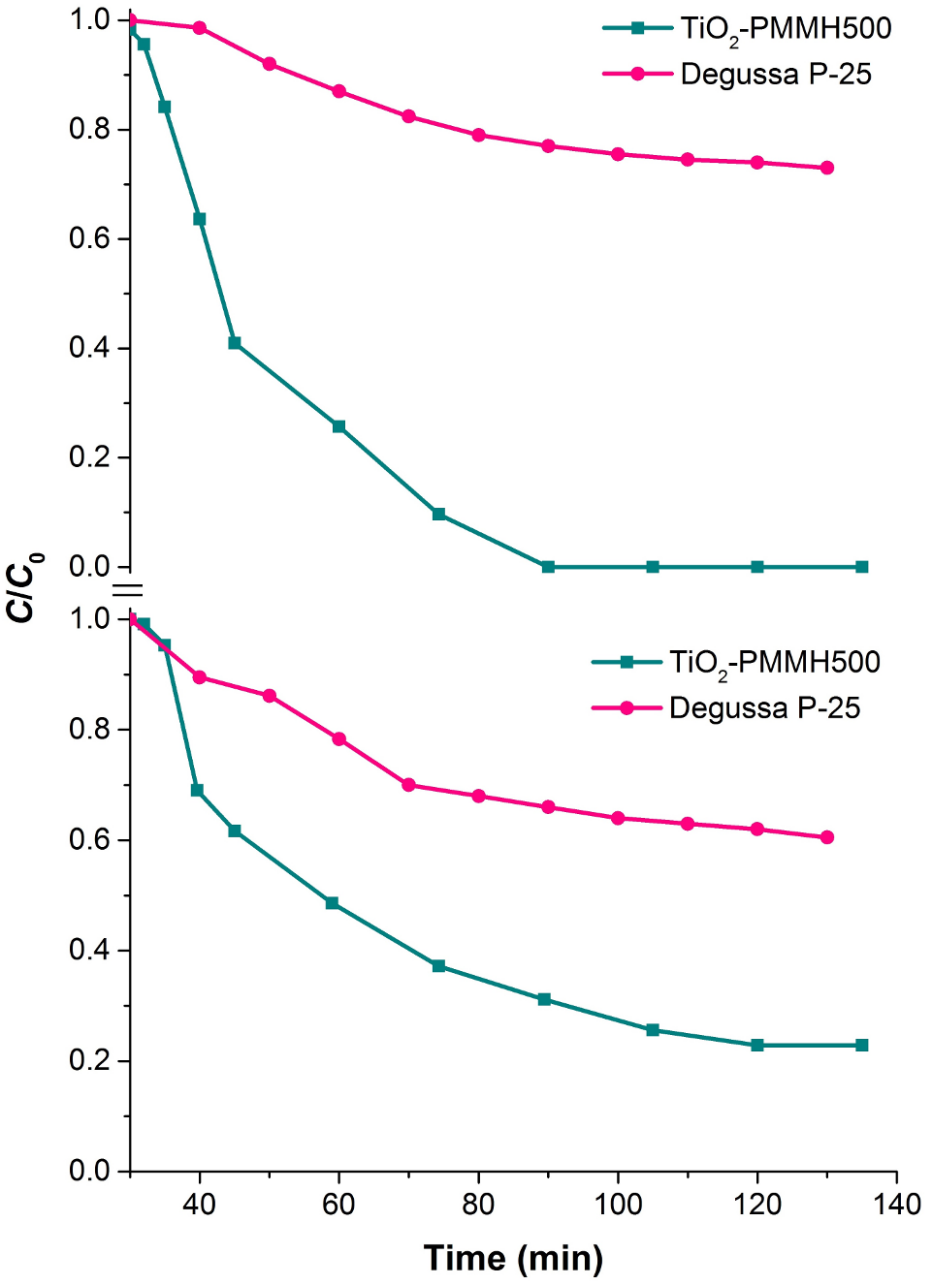
Comparison of photocatalytic activity of TiO_2_-pillared MM with the commercial photocatalyst Degussa P25 upon degradation of methyl orange (MO) and rhodamine B (RhB) dyes in aqueous solution.

Figure 12 compares the effectiveness of photocatalytic decomposition of anionic and cationic dyes at the best samples of pillared materials obtained in this work, as well as on the commercially available photocatalyst Degussa P25. The synthesized samples show significantly higher photocatalytic activity.

## Conclusion

TiO_2_-pillared MM was obtained by hydrothermally intensified intercalation of titanium polyhydroxo complexes, i.e., products of TiCl_4_ controlled hydrolysis. The porous structure of the material is thermally stable due to polymerization of titanium polycations and aggregation of pillars in the interlayer space of MM, as evidenced by high values of basal distance *d*_001_ and total pore volume. For the hydrothermal treatment it was found that: (1) the pillars of TiO_2_, representing a mixture of anatase and rutile phases, possessed a higher degree of crystallinity, and their size is confined to the range 5.6 to 12.7 nm; (2) the highest efficiency in the processes of adsorption and photocatalysis for water-soluble dyes of anionic and cationic types is observed for the samples annealed at 500 °C – a combined adsorption and photocatalytic method made it possible to remove 100% MO and 97.5% RhB in about 100 minutes. The kinetics of dye adsorption on all pillared materials under study is amenable to the pseudo-first order kinetic model. The electrostatic interaction (negative charge of modified clay particles) was found to be related to a high adsorption capacity in removing cationic dyes as compared to anionic ones. The pillared materials prepared using hydrothermal treatment combine sorptive and photocatalytic properties and can be recommended for mineralization and decomposition of organic contaminants, even in highly acidic media.

## Experimental

### Montmorillonite

A fraction of MM particles with a size of <2 micrometers, which was isolated by the sedimentation method from natural bentonite of the Dash-Salakhly deposit [56], was used as a starting material. The extracted MM fraction was subjected to drying at a temperature of 60 °C.

**Figure 13:**
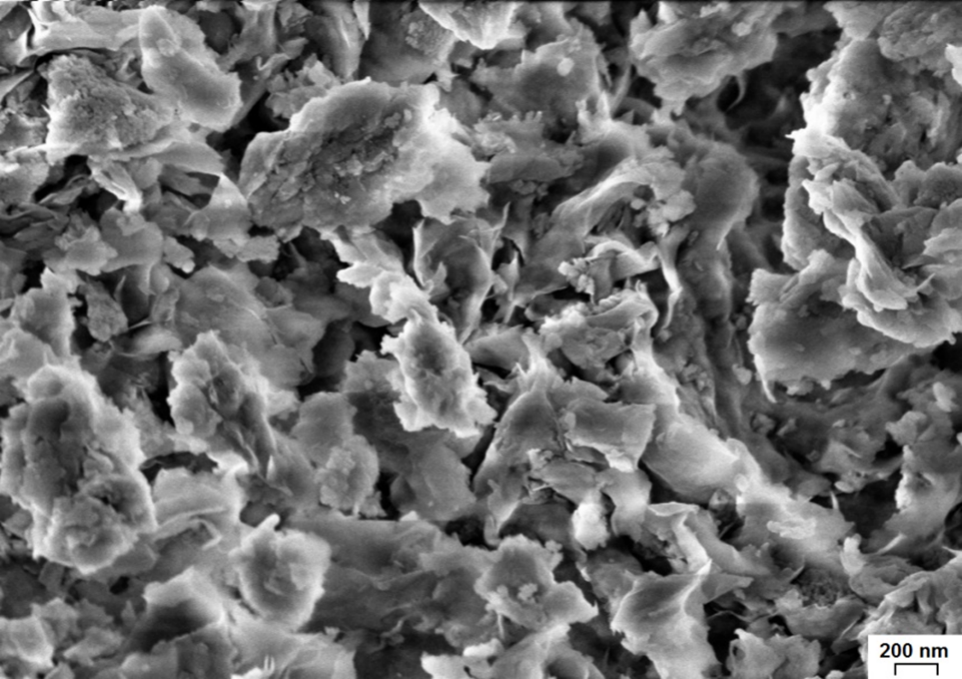
SEM image of the surface of original MM.

Original MM (Figure 13) has a characteristic layered structure and consists of flaky particles with sizes ranging from 100 to 1000 nm, which "stick" to large aggregates.

A MM form enriched with the Na^+^ ions was obtained by adding a 1 M solution of NaCl to the MM suspension (10 g of MM per 1 L of solution) under continuous mixing with a magnetic stirrer and heating to 80 °C for 2 hours. The resulting suspension was washed to remove the Cl^−^ ions with distilled water until a negative reaction with AgNO_3_ was achieved, then centrifuged and dried at a temperature of 60 °C.

### Intercalation solutions

Solutions containing titanium polycations were prepared at room temperature by hydrolyzing titanium chloride according to a procedure from [9]. To this end, a 6 M solution of HCl was treated drop wise with TiCl_4_ (Sigma-Aldrich) to obtain solutions with Ti^4+^ concentrations of 4.92 M (solution concentration of 4.92 M is the upper limit of sol formation). For further intercalation the solutions were diluted by slowly adding deionized water to yield solutions with a residual Ti^4+^ concentration of 0.56 M. Prior to use the intercalation solutions were aged for 3 hours at 20 °C, resulting in the formation of titanium polyhydroxo complexes.

**Figure 14:**
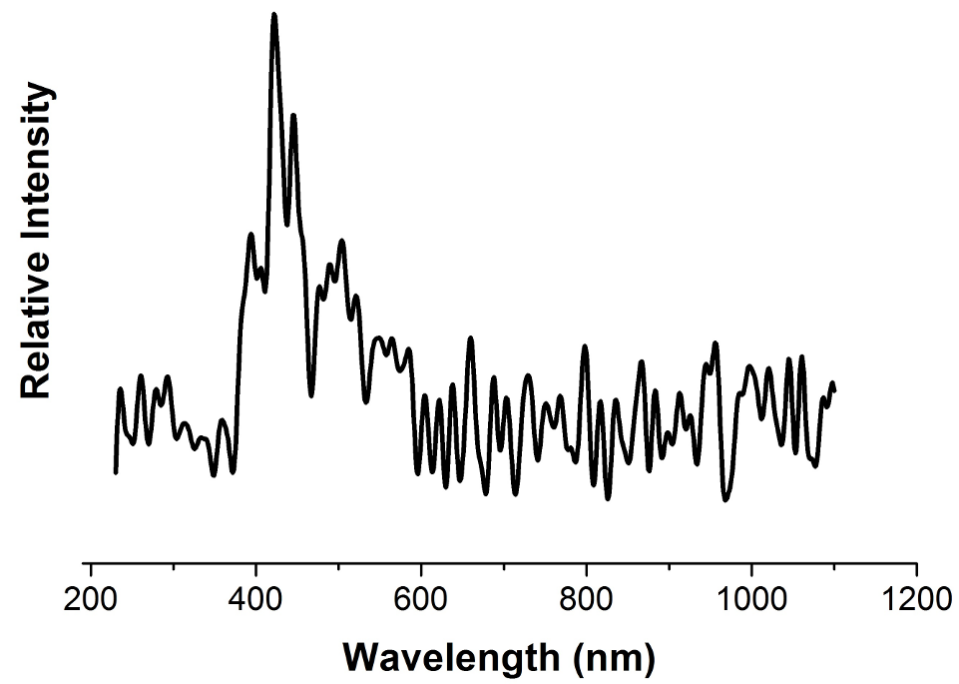
The fluorescence spectrum of an intercalating solution of titanium polyhydroxo complexes.

Figure 14 shows the fluorescence spectrum of an intercalation solution (scanning spectrofluorometer Varian Cary Eclipse, wavelength range 200–1000 nm, excitation wavelength 210 nm). Note that the solution spectrum has a peak at 422 nm, which apparently corresponds to polynuclear hydroxo complexes, because it disappears after fivefold dilution of the solution.

**Figure 15:**
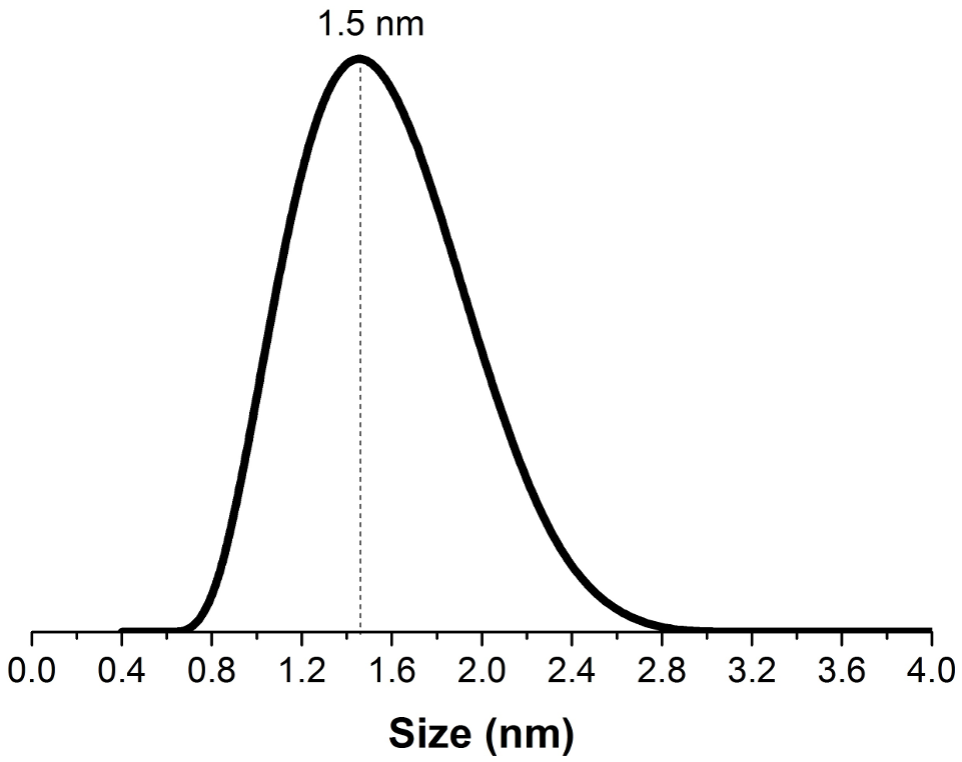
The dynamic light scattering (DLS) spectrum.

Figure 15 shows the results of particle size analysis for the intercalation solution using the laser beam dynamic scattering method (analyzer Zetasizer Nano ZS «Malvern Instruments Ltd», He–Ne laser with a wavelength of 633 nm and a recording angle of 173°). The particle size distribution curve features a peak at 1.5 nm, which corresponds to polymeric forms of the (TiO)_8_(OH)_12_^4+^ polycations, as mentioned previously [8,26].

### Intercalated and TiO_2_-pillared montmorillonite

The intercalation of montmorillonite with titanium polyhydroxo complexes was performed by ion exchange in a 1% aqueous suspension by adding the intercalating solution drop wise (10 mmol Ti^4+^/g MM) and stirring vigorously with a magnetic stirrer for 3 hours at 20 °C. After coagulation for 12 h at room temperature the suspension was washed to remove the Cl^–^ ions, centrifuged and subjected to drying in an oven at a temperature of 60 °C. The intercalated samples are designated as Ti-MM.

The effectiveness of intercalation was monitored photometrically (spectrophotometer UV-VisU-2001, Hitachi, Japan), with photometric technique based on the formation of a complex titanium(IV) compound with hydrogen peroxide, yellow in color, which was studied at a wavelength of 400–450 nm) determining the total concentration of the Ti^4+^ ions in polycations before and after completion of intercalation. For the applied solution of titanium hydroxo complexes it is found that prior to intercalation the concentration of Ti^4+^ ions was 1075 mg/L, and after that a substantial decrease in their concentration (to 700 mg/L) was observed. The amount of the Ti^4+^ ions in the interlayer space of MM was 187.5 mg/g of MM.

When using hydrothermal treatment at the MM intercalation stage the preparation of intercalating solution and the clay modification process were identical to those described above. In this case the MM and intercalating solution suspensions were subjected to hydrothermal treatment for 5 h at a temperature of 115 °C and a pressure of 170 kPa using a pressurized reactor with teflon tube. The samples intercalated using hydrothermal treatment are designated as Ti-MMH.

The pillared materials were obtained by annealing the samples in an oven at temperatures of 300, 500, 700, and 800 °C for 3 h. Samples of pillared materials without treatment are denoted as TiO_2_-PMM*x*, and those subjected to hydrothermal treatment at the intercalation stage as TiO_2_-PMMH*x*, where *x* is the calcination temperature.

### Sample characterization

XRD analysis and measurements of the basal distance *d*_001_for the samples by small-angle diffraction was performed using a Bruker D8 Advance X-ray diffractometer (Bruker-AXS, Germany). The average crystallite size (*L*) of anatase was evaluated using Scherer’s equation:

[8]
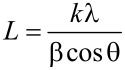


where *k* is the dimensionless coefficient of particle shape (0.94), λ is the X-ray wavelength, β is the width of reflex at half height (in units of 2θ), θ is the diffraction angle.

IR spectra of TiO_2_-pillared materials were recorded at room temperature using a Fourier transform IR spectrophotometer 360 Avatar ESP in the wavenumber range 400 to 4000 cm^−1^ with a resolution of 2 cm^−1^ and averaging over 64 scans.

A synchronous thermal analysis was performed using a NETZSCH STA 449F3 Jupiter (Netzsch, Germany) at a sample heating rate of 5 K/min.

Porosimetric measurements were carried out by low-temperature nitrogen adsorption–desorption using a specific surface area and porosity analyzer ASAP 2020 (Micromeritics, USA); prior to measurements the samples were degassed at 180 °C and residual pressure of 5–10 Pa for 3.5 h.

An analysis of the sample surface morphology was performed using an IEK-2 scanning electron microscope (Zeiss SUPRA 50VP, Germany) by coating a conductive layer of iridium. Elemental analysis of the surface was carried out on a VEGA 3 SBH scanning electron microscope integrated with a Bruker energy-dispersive microanalyzer.

### Photocatalytic activity measurements

The photocatalytic activity of the produced samples of TiO_2_-pillared MM was evaluated by studying degradation rates for dyes of anionic and cationic types, i.e., methyl orange (MO) and rhodamine B (RhB) in aqueous solution. The source of UV irradiation was a high pressure mercury lamp rated at 250 watts with an emission maximum at 365 nm. The lamp was located in a water cooled quartz jacket placed in the center of the reaction vessel with a volume of 800 mL. The bottom of the reactor featured a magnetic stirrer, which ensured effective mixing of the reaction mass. The reaction solution was purged with air at a constant rate to provide a constant concentration of dissolved oxygen therein. In each experiment a dye solution (500 mL) with a concentration of 40 mg/L was treated by synthesized photocatalyst powder in an amount of 1 g/L. The reaction mixture was stirred for a predetermined time (0 to 135 min) at 25 °C. After certain time intervals 1 mL of the suspension was picked. Then, the dye solution was separated from the photocatalyst by centrifugation at 8000 rpm for 15 min. Amount of dye in the solution after centrifugation was determined with a UV-VisU-2001 spectrophotometer (Hitachi, Japan) by measuring absorbance at a wavelength corresponding to the maximum of the absorption spectrum for MO (λ_max_ = 505 nm) and RhB (λ_max_ = 554 nm). Preliminary irradiation of dye solutions for 1 h in the absence of photocatalysts showed that no significant decoloration changes occurred in the meantime.

The study of the adsorption behavior of MO on the obtained samples of photocatalysts was carried out under the same conditions as in the investigation of their photocatalytic activity, but without UV irradiation and air purge of the reaction solution. An amount of adsorbed dye (*q*_t_, mg/g) on the sample at the time *t* was calculated by the equation:

[9]
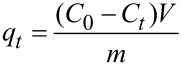


where *C*_0_ and *C_t_* (mg/L) are the initial concentration of dye and the dye concentration at time *t* (min), *V* is the volume of the dye solution (L), *m* is the mass of air-dried adsorbent (g).
